# EphA2 Is a Clinically Relevant Target for Breast Cancer Bone Metastatic Disease

**DOI:** 10.1002/jbm4.10465

**Published:** 2021-03-09

**Authors:** David B Vaught, Alyssa R Merkel, Conor C Lynch, James Edwards, Mohammed Noor Tantawy, Timothy Hilliard, Shan Wang, Todd Peterson, Rachelle W Johnson, Julie A Sterling, Dana Brantley‐Sieders

**Affiliations:** ^1^ Department of Cancer Biology Vanderbilt University School of Medicine Nashville TN USA; ^2^ Vanderbilt Center for Bone Biology Vanderbilt University School of Medicine Nashville TN USA; ^3^ Vanderbilt‐Ingram Cancer Center Vanderbilt University School of Medicine Nashville TN USA; ^4^ Department of Tumor Biology H. Lee Moffitt Cancer Center Tampa FL USA; ^5^ Botnar Research Centre University of Oxford Oxford UK; ^6^ Radiology and Vanderbilt Institute of Imaging Sciences Vanderbilt University School of Medicine Nashville TN USA; ^7^ Department of Medicine, Division of Rheumatology and Immunology Vanderbilt University School of Medicine Nashville TN USA; ^8^ Division of Clinical Pharmacology Vanderbilt University School of Medicine Nashville TN USA; ^9^ Department of Veterans Affairs, Tennessee Valley Healthcare System (VISN 9) Vanderbilt University Nashville TN USA

**Keywords:** ANIMAL MODELS, BONE CELLS, CANCER, OSTEOCLASTS, PRECLINICAL STUDIES, THERAPEUTICS, TUMOR‐INDUCED BONE DISEASE

## Abstract

EphA2 receptor tyrosine kinase (RTK) is highly expressed in breast tumor cells across multiple molecular subtypes and correlates with poor patient prognosis. In this study, the potential role of EphA2 in this clinically relevant phenomenon is investigated as metastasis of breast cancer to bone is a major cause of morbidity and mortality in patients. It was found that the EphA2 function in breast cancer cells promotes osteoclast activation and the development of osteolytic bone disease. Blocking EphA2 function molecularly and pharmacologically in breast tumors reduced the number and size of bone lesions and the degree of osteolytic disease in intratibial and intracardiac mouse models, which correlated with a significant decrease in the number of osteoclasts at the tumor–bone interface. EphA2 loss of function in tumor cells impaired osteoclast progenitor differentiation in coculture, which is mediated, at least in part, by reduced expression of IL‐6. *EPHA2* transcript levels are enriched in human breast cancer bone metastatic lesions relative to visceral metastatic sites; EphA2 protein expression was detected in breast tumor cells in bone metastases in patient samples, supporting the clinical relevance of the study's findings. These data provide a strong rationale for the development and application of molecularly targeted therapies against EphA2 for the treatment of breast cancer bone metastatic disease. © 2021 The Authors. *JBMR Plus* published by Wiley Periodicals LLC. on behalf of American Society for Bone and Mineral Research.

## Introduction

Metastasis to bone is a common occurrence among late‐stage breast cancer patients,^1^, ^2^ and approximately 70% of patients who die from breast cancer have bone metastases.^3^ Breast‐to‐bone metastases are predominately osteolytic in nature and cause skeletal lesions that result in fractures, nerve compression, bone pain, and hypercalcemia.^4^, ^5^, ^6^, ^7^ The establishment and growth of these metastases depend on the interaction between tumor cells and the host microenvironment. Metastatic cells are able to seize control of molecular pathways that regulate normal bone remodeling to induce aberrant activation of osteoclasts, which leads to increased lysis of the bone.^5^ Osteoclasts, multinucleated differentiated cells with the unique ability to resorb mineralized bone, are critical for tumor‐induced osteolysis.^8^ Identifying molecular regulators of osteoclast recruitment, maturation, and activation is key for developing new molecularly targeted therapies to inhibit bone destruction of osteolytic lesions resulting from breast cancer metastasis to the bone.

Recent studies suggest Eph receptor interactions with ephrin ligands mediate bone homeostasis in both A and B subclasses.^9^, ^10^ Cell surface‐bound ephrin ligands and their receptors (Eph) belong to the largest family of receptor tyrosine kinases. The Eph family of receptors and ligands plays critical roles in neuronal, vascular, and intestinal development, as well as cellular migration and bone morphogenesis.^11^, ^12^, ^13^, ^14^, ^15^, ^16^, ^17^ Both ephrin ligands and Eph receptors are membrane‐bound proteins, which generate signaling via cell–cell contact in both the receptor (forward signaling) and ligand–(bidirectional/reverse signaling) expressing cells. The family is subdivided into two subclasses based on sequence homology, binding affinity, and structure. The A subclass of receptors (EphA1‐EphA10) generally bind to the ligands tethered to the cell membrane by a glycosylphosphatidylinositol (GPI) anchor (ephrinA1‐ephrinA6), whereas the B‐subclass (EphB1‐EphB4, EphB6) generally bind to ligands containing a transmembrane domain followed by a short cytoplasmic region (ephrinB1‐ephrinB3). The importance of signaling by both the receptor and ligand has been confirmed in multiple studies of angiogenesis, tissue boundary formation, cell sorting, and axonal guidance.^18^

A‐class Eph receptors have also been implicated in bone homeostasis. Irie and colleagues have shown that ephrinA2‐EphA2–mediated interactions between osteoclast precursors and osteoblasts enhance osteoclastogenesis while inhibiting osteoblast differentiation.^10^ Likewise, other studies have implicated A‐class receptors in giant cell tumors^19^ and prostate cancer metastasis.^20^, ^21^, ^22^ Several studies have linked EphA2 function to breast tumor growth and visceral metastasis in multiple breast cancer subtypes,^23^ including HER2+^24^ and basal‐like, triple‐negative models.^25^ These studies are consistent with enriched *EPHA2* transcript levels and protein expression in these subtypes in human breast cancer, with higher levels of *EPHA2* correlating with poor clinical outcome.^25^, ^26^ Recently, a clinical trial testing a combination therapy with dasatinib, which cotargets Src family kinases and EphA2 along with other kinases, plus zolendronic acid, a bisphosphonate that inhibits osteoclast proliferation, produced responses in hormone receptor–positive metastatic breast cancer with skeletal involvement.^27^ Although EphA2 receptor tyrosine kinase plays a crucial, clinically relevant role in breast cancer growth across multiple subtypes and in visceral metastasis,^23^, ^24^, ^25^, ^26^, ^28^, ^29^, ^30^ its role in breast‐to‐bone metastasis remains unclear.

Here, we show that the EphA2 function in breast cancer cells promotes osteoclast activation and the development of osteolytic bone disease. *EPHA2* transcript levels were elevated in human breast‐to‐bone metastases, and EphA2 loss of function in tumor cells reduced tumor‐induced osteolysis in two independent models in vivo. Further analyses revealed that blocking tumor EphA2 function reduced osteoclast precursor maturation into functional osteoclasts through an IL‐6–dependent mechanism in coculture. Together, these data provide preclinical validation of EphA2 as a clinically relevant molecular target for breast cancer bone metastatic disease, warranting further investigation of molecular mechanisms that link EphA2 to tumor‐induced osteolysis and potential development of a clinical inhibitor.

## Materials and Methods

### Reagents

Raw246.7 cells were purchased from the American Type Culture Collection (ATCC). The generation of 4T1 control (4T1.V) and 4T1 dominant–negative EphA2‐overexpressing cells (4T1.ΔC) was described previously.^29^ The generation of MDA‐MB‐231 vector control and EphA2 shRNA knockdown (KD) cells was described previously.^25^ EphA2 antibodies were purchased from Santa Cruz Biotechnology (SC‐924), Millipore/Sigma (Clone D7), Thermo Fisher Scientific (34–7400), and Cell Signaling Technology (P‐EphA2 Y588 D7X2L mAb). Stat3 antibodies were purchased from Cell Signaling Technology (Stat3 D1B2J mAb; P‐Stat3 Y705 D3A7 mAb). Actin antibodies were purchased from Santa Cruz Biotechnology (SC‐1616 and SC‐47778). TaqMan qRT‐PCR reagents were purchased from Thermo Fisher Scientific. Primer sequences for qRT‐PCR: *EphA2‐F* CCCCGCCCCTAGTTAGAGG; *EphA2‐R* GAAAGCAAGAAGCTGGCCC; *GAPDH‐F* AACTTTGGCATTGTGGAAGG; *GAPDH‐R* ACACATTGGGGGTAGGAACA. ELISA kits for mouse and human IL‐6 were purchased from RayBiotech. Neutralizing anti‐mouse IL‐6 antibody (AF‐406‐NA), recombinant mouse and human IL‐6, and recombinant mouse ephrin‐A1‐Fc were purchased from R&D Systems. Neutralizing anti‐human IL‐6 antibody was purchased from Abcam. Recombinant murine RANK ligand was purchased from Peprotech. All other reagents were obtained from Sigma‐Aldrich unless otherwise noted. Tissue processing and histologic analyses were performed by the Vanderbilt Translational Pathology Shared Resource (TPSR) or the Vanderbilt Center for Bone Biology.

### Mining the Human Cancer Metastasis database

The Human Cancer Metastasis database^31^ (http://hcmdb.i-sanger.com/index) was queried for “EphA2” in two independent breast metastatic datasets (GSE14017 and GSE14020) to compare relative transcript expression in bone relative to brain, lung, and liver metastatic sites. For statistical analyses within the database, the limma package nested in R (http://www.r-project.org/) was employed to detect differentially expressed genes between different types of samples in the database. Those genes with false discovery rate (F.D.R.)<0.05 were selected as candidates having significantly different expressions. *EPHA2* was identified as having a significantly different expression between bone metastases relative to lung, brain, and/or liver.^31^ Human samples from the Human Cancer Metastasis database and the human breast‐to‐bone metastasis sections were de‐identified and were thus exempt from institutional review board authorization.

### qRT‐PCR and Western blot analyses

qRT‐PCR for *EPHA2* in MDA‐MB‐231 vector versus EphA2 shRNA KD was performed as described previously.^32^ Western blot detection of P‐EphA2 and/or total EphA2 in 50 μg of cell lysate (4T1 and MDA‐MB‐231) was performed as described previously.^24^ To validate recombinant mouse or human IL‐6 and neutralizing anti‐mouse or anti‐human IL‐6, RAW264.3 cel1s (2 × 10^5^) were seeded in six‐well plates, starved overnight in OptiMem media, and stimulated for 5 min with IL‐6 (50 ng/ml) that had been preincubated for 30 min with control IgG (20 μg/ml mouse or goat; Santa Cruz Biotechnology) or neutralizing anti‐IL‐6 antibody (20 μg/ml). Lysates were harvested and 50 μg was fractionated, subjected to SDS‐PAGE and transfer, and membranes probed with anti‐P‐Stat3 and anti‐Stat3 antibodies. Membranes were probed with anti‐actin antibodies to confirm uniform loading.

### Intratibial and intracardiac injections

All experiments involving animals were performed in accordance with the Association for Assessment and Accreditation of Laboratory Animal Care (AAALAK International guidelines and with Vanderbilt University Institutional Animal Care and Use Committee approval. Animals were housed in a pathogen‐free facility, including sterile food, water, and bedding for nude mice. General health and weight of experimental animals was monitored two to three times weekly for the duration of each experiment. 4T1.V and 4T1ΔC tumor cells (10^5^ cells in a 25 μl volume of sterile PBS) were injected into the left tibia of deeply anesthetized 6‐ to 8‐week‐old BALB/c female mice (Envigo). The contralateral tibia was injected with 25‐μl volume of PBS alone and treated as the sham‐injected control. Mice were euthanized 10 days postsurgery, and both the tumor‐injected and contralateral tibias were collected for histologic analyses. The same number/volume of vector control or EphA2 shRNA KD MDA‐MB‐231 cells were injected into anesthetized immunocompromised 6‐week‐old nude female mice (Envigo). Tibias from both the tumor‐injected and sham contralateral legs were also harvested for analysis 3 weeks postimplantation. Intracardiac injection (left ventricle) of 1 × 10^5^ MDA‐MB‐231 cells in 100 μl PBS into 6‐week‐old nude female mice was performed as described previously.^33^ Hindlimbs were collected for histologic analyses 3 weeks postinjection. Mice were monitored two to three times per week for adverse effects, including difficulty ambulating and cachexia. All animal studies were repeated at least twice for a total of 8‐12 animals per condition.

### Treatment with ALW‐II‐41‐27

Mice (6‐week‐old nude female) harboring MDA‐MB‐231 tumors (intracardiac injection of 1 × 10^5^ MDA‐MB‐231 cells) were randomized and treated with 15 mg/kg ALW‐II‐41‐27 EphA2 inhibitor in 10% 1‐methyl‐2‐pyrrolidinone and 90% polyethylene glycol 300 or the vehicle starting 1 week after tumor cell injection. Mice were treated twice daily (intraperitoneal injection) for 5 days with a 2‐day holiday in between over the course of 2 weeks, and were imaged at week 1 posttreatment and week 2 posttreatment by Faxitron digital X‐ray (see below) for analysis and quantification of osteolytic bone disease prior to collection of hindlimbs for histology. Mice were monitored two to three times per week for adverse effects, including difficulty ambulating and cachexia; there were seven animals per condition.

### Histology

#### Tartrate‐resistant acid phosphatase staining

Tibias and femurs were fixed for 24 hours in fresh 10% neutral buffered formalin, decalcified, embedded in paraffin, and sectioned by the Vanderbilt Center for Bone Biology. Tartrate‐resistant acid phosphatase (TRAP) staining on tissue sections was performed by the Vanderbilt Center for Bone Biology as described previously.^34^ Multinuclear TRAP+ osteoclasts adjacent to bone were quantified using cellSens (Olympus) morphometric software and normalized to the bone area. For coculture studies, TRAP staining was performed using Sigma‐Aldrich TRAP staining kit as per the manufacturer's instructions. Multinuclear TRAP+ osteoclasts were quantified by hand‐counting and by cellSens morphometric software. The pixel area of the multinuclear TRAP+ osteoclasts was measured using ImageJ software (National Institutes of Health; https://imagej.nih.gov/ij/).

#### EphA2 staining

Immunohistochemical detection of EphA2 in paraffin sections from collected hindlimbs was performed as described previously,^35^ using a rabbit polyclonal antibody (5 μg/ml, overnight at 4°C; Zymed Laboratories) validated in human tumor sections and using EphA2‐deficient mice.^36^ Antigen retrieval was performed by heating in a Pickcell 2100 retriever (PickCell Laboratories BV) in the presence of citrate buffer (2mM citric acid, 10mM sodium citrate buffer, pH 6.0). Sections were washed in PBS and incubated with primary antibody overnight, followed by biotinylated anti‐rabbit IgG secondary antibody (1:200; Transduction Laboratories, BD Biosciences PharMingen) for 1 hour at room temperature. Specific staining was detected using avidin‐peroxidase (ABC kit; Vector Laboratories) followed by 3,3′ diaminobenzidine substrate (Zymed Laboratories). Sections were counterstained with hematoxylin. Immunohistochemical detection of EphA2 from human breast‐to‐bone metastasis samples (de‐identified and generously provided by Dr Conor Lynch) was performed as described above.

#### Ki67 and cleaved caspase 3

Staining was performed by the Vanderbilt University Medical Center TPSR. Proliferation and apoptosis indices were calculated as described previously.^25^

### Faxitron analysis

At weeks 1‐3 posttransplantation, the mice were anesthetized with 2% isoflurane and imaged in a Faxitron LX‐60 (Faxitron Biooptics) at an X‐ray energy of 35 kVp for 8 s. Visual inspection of the two‐dimensional digital X‐ray images was carried out to identify and enumerate lesions, and lesion areas were determined using ImageJ software.

### μComputed tomography and histomorphometry

At week 8 postintratibial injection, the animals were anesthetized with 2% isoflurane and imaged in a microCAT II (Siemens) at an X‐ray beam intensity of 180 mAs and an X‐ray tube voltage of 80 kVp. The images were reconstructed at 512 × 512 × 512 with a voxel size of 0.122 × 0.122 × 0.173 mm^3^. Three‐dimensional bone segmentation and quantitation of osteolytic lesions of the reconstructed images were carried out using the imaging software Amira (Thermo Fisher Scientific). The animals were euthanized at week 8 based upon μCT scan analysis. After euthanization, the samples were fixed for 24 hours in 10% formalin and decalcified as described previously.^34^ Tissues were embedded in paraffin, and 5‐μm sections were prepared for staining. Histomorphometry was calculated by using two nonserial H&E‐stained sections of tumor–bearing limbs to assess bone volume/total volume and/or with TRAP to provide osteoclast number per millimeter of bone at the tumor bone interface.

### Cytokine array and ELISA

Quantibody Mouse Cytokine Array (QAM‐CYT‐Q‐2000) was purchased from RayBiotech and used to analyze conditioned medium harvested from 4T1.V and 4T1.ΔC lines as per the supplier's instructions. To generate conditioned media, 3 × 10^6^ cells were plated in 10‐cm tissue culture dishes and incubated in serum‐free OptiMem for 48 hours prior to collection. Conditioned medium was filtered to remove cell debris and particulates prior to analysis. To validate protein expression profiles for IL‐6, conditioned media from 4T1.V and 4T1.ΔC was harvested and analyzed using a mouse IL‐6 ELISA kit (RayBiotech) as per the manufacturer's instructions. Conditioned media from MDA.V and MDA.A2KD cells were analyzed using a human IL‐6 ELISA kit (RayBiotech).

### Cell culture

4T1 and MDA‐MB‐231 were maintained in DMEM supplemented with 10% FBS as described previously.^37^ RAW264.7 cells were maintained in αMEM supplemented with 10% FBS. For some experiments, RAW264.4 cells were differentiated into osteoclasts by stimulation with 50 ng/ml RANKL (Peprotech).

### Coculture

RAW264.7 cells (1 × 10^4^) were plated on the bottom of a 24‐well transwell plate and grown in α‐MEM/10% FBS. Transwell inserts (0.4‐μm pore size; Costar) containing 5000 tumor cells (4T1.V vs 4T1.ΔC and MDA‐MB‐231 vector vs EphA2 KD) with and without EphA2 function and cultured for 5 days. TRAP staining assays were performed to detect activated osteoclasts in vitro according to the manufacturer's instructions. For some experiments, recombinant IL‐6 (50 pg/ml in  4T1.ΔC and MDA.A2KD cocultures to restore IL‐6 expression to control  4T1.V levels) or neutralizing anti–IL‐6 antibody (20 μg/ml in  4T1.V and MDA.V cocultures to block IL‐6 function) was added to transwells and chambers during culture. PBS vehicle and control goat or mouse IgG were used as negative controls. Untreated RAW264.7 cells served as negative controls, and RAW264.7 cells treated with 50 ng/ml RANKL were used as positive controls for osteoclast differentiation. For direct coculture, RAW264.7 cells (1 × 10^4^) were plated in six‐well plates and grown in α‐MEM/10% FBS for 24 hours. Tumor cells (5000  4T1.V vs 4T1.ΔC and MDA.V vs MDA.A2KD) were added and cultures were maintained for 4 days. TRAP staining assays were performed as described above. For some experiments, recombinant IL‐6 or neutralizing anti‐IL‐6 antibodies were used to restore expression to normal levels (4T1.ΔC and MDA.A2KD) or block IL‐6 activity in control cells (4T1.V and MDA.V) as described above. Direct cocultures between  4T1 and primary mouse bone marrow cells were performed to confirm data derived from the RAW264.7 coculture model. Briefly, mouse bone marrow was isolated from 5‐week‐old BALB/c female mice. Following red blood cell lysis, cells were suspended in α‐MEM/10% FBS and plated on tissue culture plastic. After 2 hours, nonadherent cells were collected and seeded into 24‐well plates (2 × 10^6^ cells) and cultured overnight. After 24 hours, tumor cells (1.000  4T1.V vs 4T1.ΔC) were added plus or minus recombinant IL‐6 or neutralizing anti‐IL‐6 antibodies as described above. Cocultures were maintained for 8 days prior to TRAP staining as described above.

### Statistical analysis

All graphs are mean ± standard error of the mean. Statistical significance was determined by Mann–Whitney tests or ANOVA, as noted in the figure legends, using Prism 6 software (GraphPad). Statistical analyses for the Human Cancer Metastasis database was performed in the system as described.^31^ For animal experiments, the primary endpoint for experiments in which tumors were analyzed was osteolytic lesion size. We anticipated that a 40% difference in tumor volume would be biologically meaningful, thus *N* = at least 7 animals per group would provide 80% power to detect a 40% difference with a SD of 0.25 at *p* = 0.05.

## Results

### EphA2 expression is enriched in tumor cells in human breast cancer bone metastatic lesions

**Fig 1:**
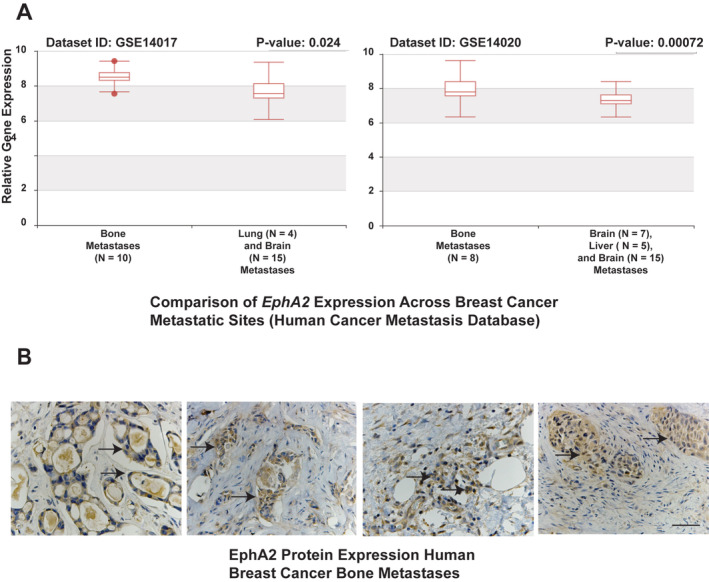
EphA2 is highly expressed in human breast‐to‐bone metastatic lesions. (*A*) *EPHA2* transcript levels in breast cancer metastatic lesions within bone relative to lung and brain (left), and in bone metastatic lesions relative to brain, liver, and lung in an independent dataset (right, *p* < 0.05). (*B*) Immunohistochemistry (IHC) staining for EphA2 was performed on human breast cancer bone metastatic samples. There were six independent human breast‐to‐bone samples for IHC staining, five of which were positive for EphA2 expression in tumor cells (arrows). Scale bar = 50 μm.

To assess the potential role of EphA2 function in the context of breast cancer metastatic disease in the bone microenvironment, we first analyzed and compared *EPHA2* transcript levels in human metastatic breast cancer patient datasets from bone, brain, and lung curated by the Human Cancer Metastasis database (http://hcmdb.i-sanger.com/index).^31^ Transcript levels of *EPHA2* were significantly higher in breast cancer bone metastases relative to other metastatic sites including brain, lung, and liver in two independent datasets (Fig. 1*A*; *p* < 0.05). Immunohistochemical staining for EphA2, using an antibody validated in human tumor samples and in EphA2‐deficient mice,^36^ was performed in a set samples from de‐identified patients with human breast cancer bone metastases (generously provided by Dr Conor Lynch). This analysis revealed high levels of EphA2 protein expression in tumor cells (Fig. 1*B*; arrows). We observed 80%‐95% EphA2+ tumor cells in these samples. These data suggest that EphA2 might play a role in breast cancer bone metastatic progression.

### Loss of EphA2 function in breast tumor cells impairs osteolysis when grafted into bone

**Fig 2:**
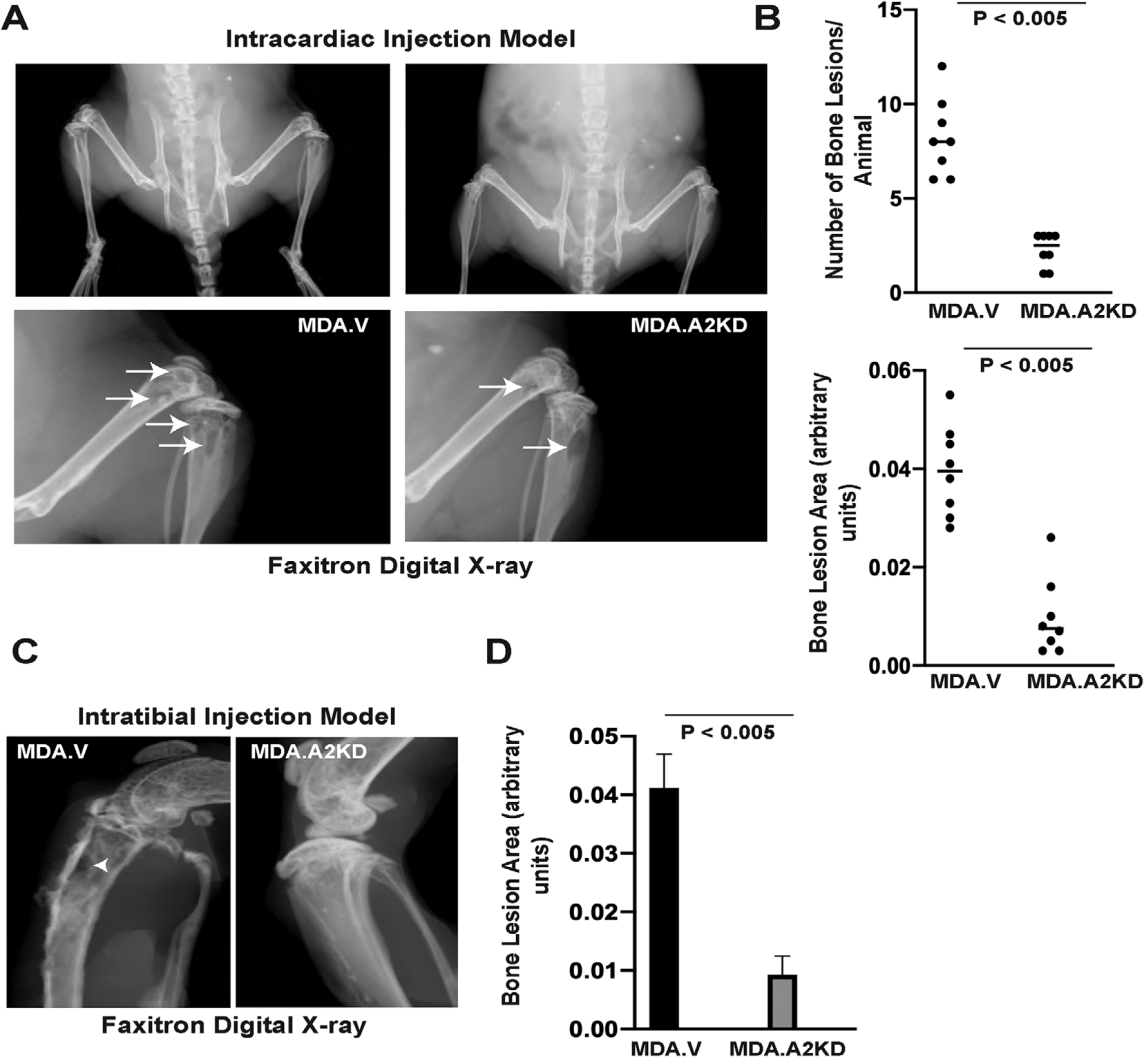
EphA2 loss of function reduces osteolytic disease in an intracardiac xenograft tumor model. (*A*) Digital X‐ray images of osteolytic lesions (arrows) 3 weeks following intracardiac injection of MDA.V or MDA.A2KD cells, which we enumerated and used morphometric software to measure the area. (*B*) Graphs show quantification of osteolytic lesion numbers (top) and sizes (bottom; *p* < 0.005, Mann–Whitney test). (*C*) Digital X‐ray images of osteolytic lesions (arrowhead, *C*) 3 weeks following intratibial injection of MDA.V or MDA.A2KD. (D) Graph shows quantification of osteolytic lesion sizes (*p* < 0.005, Mann–Whitney test). There were eight animals per condition analyzed in two experiments.

To test the role of EphA2 in bone metastatic disease in vivo, we used the MDA‐MB‐231 human breast cancer cells, which express high levels of EphA2,^25^, ^38^ to model experimental metastasis.^39^ Immunohistochemical staining for EphA2 in MDA‐MB‐231 intratibial tumors revealed that EphA2 was predominantly expressed in tumor cells (Supplementary Information Fig. S1A, right panels) within bone and in associated blood‐vessel endothelium (Supplementary Information Fig. S1A, lower left panel), consistent with what we observed in human disease (Fig. 1*B*) and in our previous studies showing EphA2 expression in the majority of tumor‐associated vascular endothelium.^37^, ^40^, ^41^ For loss of function experiments, we used MDA‐MB‐231 cells stably expressing shRNAs against EphA2 (MDA.A2KD) versus vector control (MDA.V).^25^ We confirmed EphA2 KD in MDA.A2KD cells by qRT‐PCR and immunoblot analyses (Supplementary Information Fig. S1B). We performed intracardiac injection of MDA.V and MDA.A2KD, and monitored animals for osteolytic disease progression. Three weeks after injection, we analyzed Faxitron digital X‐ray images to quantify the number and size of skeletal lesions, particularly in the femurs and tibias (Fig. 2*A*, lower panels, arrows). Quantification of the number of osteolytic lesions revealed a significant reduction in MDA.A2KD‐inoculated animals relative to MDA.V controls (Fig. 2*B*; *p* < 0.005). MDA.A2KD‐inoculated animals also displayed significantly reduced osteolytic lesion area compared with the MDA.V controls (Fig. 2*B*; *p* < 0.005).

To confirm these data, we established intratibial xenografts with MDA.V versus MDA.A2KD tumor cells and analyzed the resulting bone lesions by digital X‐ray. Relative to the vector control that produced large osteolytic lesions (Fig. 2*C*, left panel, arrowhead), MDA.A2KD‐inoculated cells produced smaller osteolytic lesions (Fig. 2*C*, right panel). Quantification of the osteolytic lesion area revealed a significant reduction in osteolytic lesion size in MDA.A2KD‐inoculated animals relative to MDA.V controls (Fig. 2*D*; *p* < 0.005), suggesting that the reduced osteolysis observed in the intracardiac model is caused by the effects on tumor outgrowth in the bone and not extravasation into the bone marrow. Osteolytic disease and bone damage were detected in tumor‐bearing limbs, but not in PBS mock‐injected contralateral control limbs (Supplementary Information Fig. S1C).

**Fig 3:**
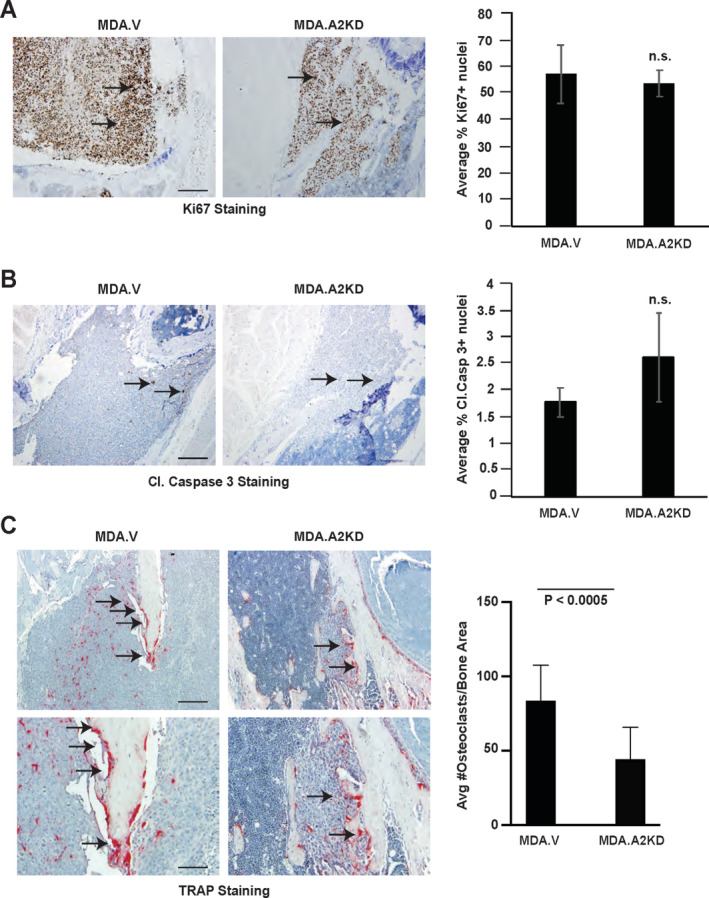
EphA2 loss of function reduces osteoclast number in the intracardiac xenograft model. (*A*) Ki67 staining (arrows) in tissue sections from MDA.V and MDA.A2KD bone tumors. Graph shows quantification of % Ki67+ nuclei relative to total nuclei in bone tumor sections (n.s. = not significant; Mann–Whitney test). (*B*) Cleaved caspase 3 (Cl. caspase 3, arrows) staining in tissue sections from MDA.V and MDA.A2KD bone tumors. Graph shows quantification of % cleaved caspase 3+ nuclei relative to total nuclei between MDA.V and MDA.A2KD tumors (n.s.; Mann–Whitney test). Tissue sections are from five independent animals per genotype, four independent ×20 fields per tumor. Scale bar = 100 μm. (*C*) Tartrate‐resistant acid phosphatase (TRAP) osteoclast staining (arrows) in tissue sections from MDA.V and MDA.A2KD bone tumors. Graph shows quantification of TRAP+ osteoclasts at the tumor–bone interface normalized to bone area (*p* < 0.005, Mann–Whitney test). There were eight animals per condition; four independent ×20 fields per tumor were analyzed in two independent experiments. Scale bar = 100 μm, upper panels; 50 μm, lower panels.

We quantified tumor cell growth and apoptosis by immunohistochemical staining for Ki67 (Fig. 3*A*, arrows) and cleaved caspase 3 (Fig. 3*B*, arrows) in bone sections from intracardiac injections. We detected no significant differences in tumor cell proliferation (Fig. 3*A*, graph, n.s. = not significant) or survival (Fig. 3*B*, graph, n.s. = not significant) in MDA.V versus MDA.A2KD tumors in bone, suggesting EphA2 loss of function does not affect tumor cell growth or survival in the bone microenvironment (Fig. 3*A*,*B*). We next quantified the number of differentiated TRAP+ osteoclasts in MDA.V versus MDA.A2KD tumors in bone (Fig. 3*C*, arrows). We observed a significant decrease in the number of TRAP+ osteoclasts proximal to bone in MDA.A2KD tumors relative to MDA.V tumors (Fig. 3*C*, graph; *p* < 0.0005), though we detected no differences in the sizes of bone proximal osteoclasts in MDA.V versus MDA.A2KD tumors (average pixel area 201.4 ± 38 MDA.V vs 204 ± 39, arbitrary units; *p* = 0.87 Mann–Whitney test), suggesting that EphA2 loss of function in tumors impairs osteoclast differentiation and/or function.

### Loss of EphA2 function in breast tumor cells results in reduced osteolytic disease and osteoclasts in an independent bone graft model

To confirm our findings in the MDA‐MB‐231 human model, we used the 4T1 mouse metastatic adenocarcinoma line as an independent model. We previously reported that an engineered mutant of EphA2 (ΔC) lacking the intracellular domain of the receptor acts as a dominant negative when overexpressed, inhibiting EphA2 activation. Overexpression of EphA2.ΔC inhibited migration and lung metastasis in vitro and in vivo in the 4T1 model.^29^ EphA2.ΔC also possesses the advantage of maintaining expression of the EphA2 extracellular domain, allowing us to disrupt forward signaling through the receptor on tumor cells, while minimizing the effects of potential bidirectional/reverse signaling through ligand‐expressing cells in the bone microenvironment. Immunohistochemical staining for EphA2 revealed that EphA2 was predominantly expressed in tumor cells within bone (Supplementary Information Fig. S2A, right panels) and associated blood vessels (Supplementary Information Fig. S2A, lower left panel), consistent with what we observed in human disease (Fig. 1*B*). Our previous studies found that EphA2 is highly expressed in tumor‐associated vascular endothelium.^42^

**Fig 4:**
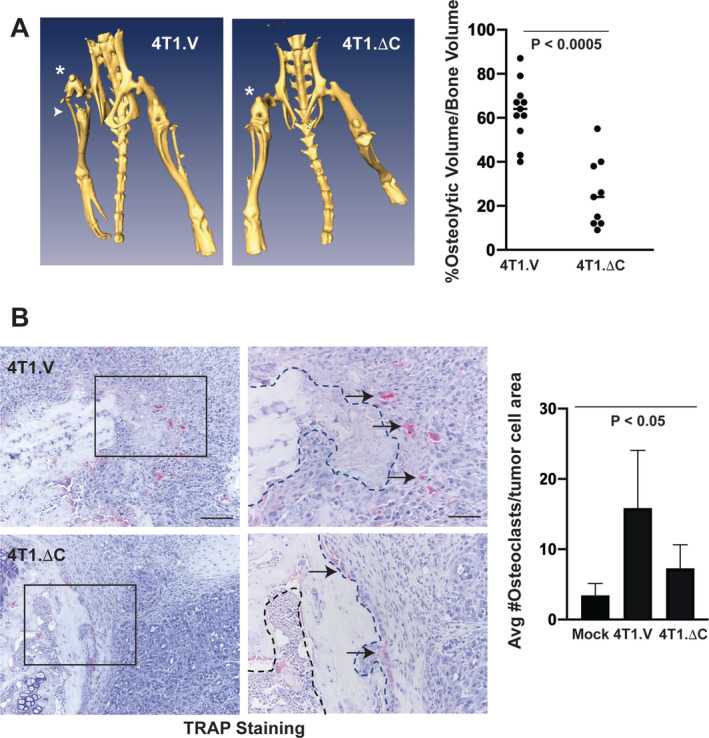
EphA2 loss of function reduces osteolytic disease in an independent intratibial allograft tumor model. (*A*) μCT for mice inoculated with  4T1 metastatic mouse mammary adenocarcinoma cells expressing a truncated EphA2 (4T1.ΔC) or vector control cells (4T1.V) and euthanized 10 days later. * Indicates tumor‐bearing limb. Arrowhead indicates area with evidence of bone destruction. Graph shows histomorphometric quantification of μCT images calculating the percentage of osteolytic volume versus bone volume in tumor‐bearing limbs (*p* < 0.0005, Mann–Whitney test). (*B*) Tartrate‐resistant acid phosphatase (TRAP) staining of osteoclasts (arrows) in tissue sections from tumor‐bearing bones, as well as PBS‐sham–injected control bones. Graph shows quantification of bone‐proximal TRAP+ osteoclasts normalized to bone area (*p* < 0.05, ANOVA). Scale bar = 100 μm, left panels. Right panels (50 μm) show higher magnification of regions marked with black boxes in left panels. Dashed lines indicate boundary between bone and tumor. There were 9 to 11 animals per condition analyzed in two independent experiments.

μCT analysis revealed osteolytic disease in the inoculated tibia for intratibial allografts of vector control (4T1.V) cells (Fig. 4*A*, left panel, arrowhead). Allograft of cells expressing dominant negative EphA2 (4T1.ΔC) showed less bone destruction in the inoculated tibia (Fig 4*A*, right panel). Quantification of the percentage of osteolytic volume relative to tumor volume revealed a significant reduction in tumor volume in the tibias of  4T1.ΔC inoculated mice (Fig. 4*A*, graph; *p* < 0.0005). Histologic analyses of tissue from tumor‐bearing animals revealed a significant reduction in the numbers of TRAP+ osteoclasts (Fig. 4*B*, arrows right panel; *p* < 0.05) proximal to bone (bone surface marked by dashed lines) for  4T1.ΔC tumors relative to vector controls, consistent with reduced osteolysis in  4T1.ΔC tumors. We confirmed the dominant negative function of EphA2 in  4T1.ΔC clones versus  4T1.V control via comparison of EphA2 receptor phosphorylation upon stimulation with soluble ligand (Supplementary Information Fig. S2B). We observed no difference in tumor cell proliferation or apoptosis in  4T1.V versus  4T1.ΔC in the intratibial model (Supplementary Information Fig. S2C,D), suggesting that EphA2 does not function to regulate tumor cell proliferation or survival in this model of bone metastatic disease.

### Targeted inhibition of EphA2 impairs osteolysis in vivo

**Fig 5:**
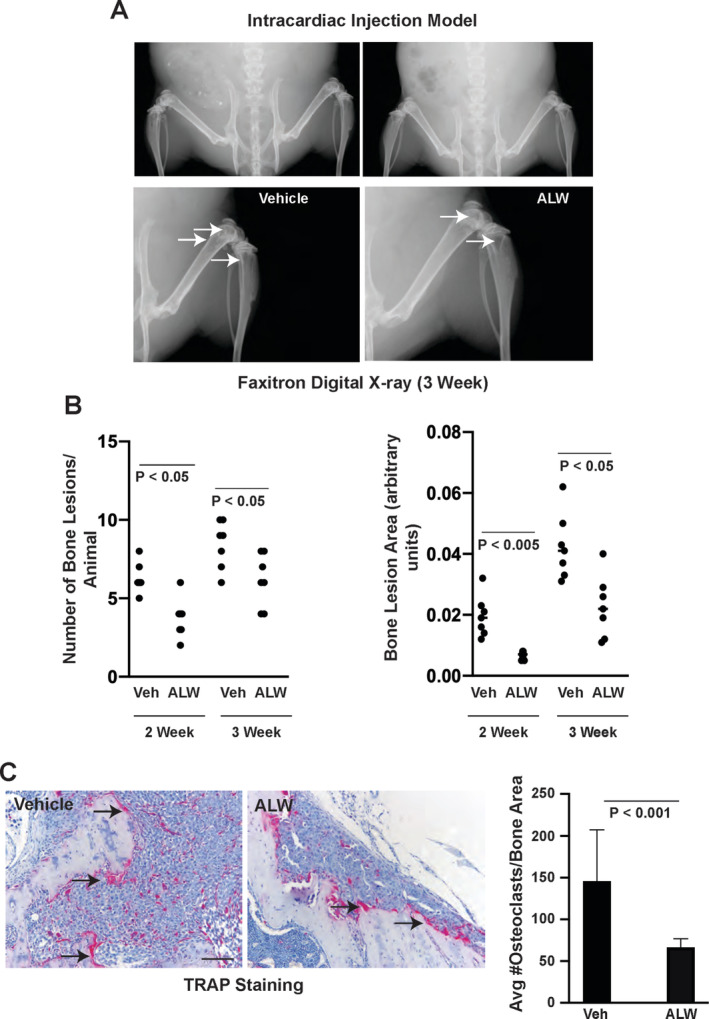
Pharmacologic inhibition of EphA2 reduced osteolytic disease in intracardiac xenograft model. One week after intracardiac injection of MDA‐MB‐321 cells into nude female mice, animals were treated twice daily with EphA2 tyrosine kinase inhibitor ALW‐II‐41‐27 (ALW) or control vehicle. (*A*) Osteolytic lesions detected in digital X‐rays (arrows) 2 weeks and 3 weeks posttreatment. (*B*) Graphs show enumeration of osteolytic lesions (left) and quantification of lesion sizes (right) by morphometric software in ALW‐treated animals versus vehicle controls (*p* < 0.05, Mann–Whitney test). (C) Tartrate‐resistant acid phosphatase (TRAP) staining of osteoclasts (arrows) in tissue sections from tumor‐bearing bones. Graph shows quantification of TRAP+ osteoclasts proximal to bone normalized to bone area (*p* < 0.001, Mann–Whitney test). Scale bar = 100 μm. There were eight animals per condition.

To investigate the efficacy of an EphA2 pharmacologic inhibitor on breast cancer–induced osteolysis, we treated bone tumor–bearing animals with EphA2 small‐molecule inhibitor ALW‐II‐41‐27^25^, ^43^ or vehicle control. Following intracardiac injection of MDA‐MB‐231 human tumor cells into nude female hosts, tumors were allowed to grow for 1 week prior to treatment with ALW versus control for 2 weeks. Osteolytic lesions were observed after 2 weeks and 3 weeks, particularly in the femurs and tibias (Fig. 5*A*, arrows, lower panels). We observed a significant reduction in both the number and size (Fig. 5*A*,B, *p* < 0.05) of skeletal lesions in ALW‐treated animals relative to controls at both 2 weeks and 3 weeks postinjection, 1 week and 2 weeks posttreatment. We did not observe significant differences in tumor cell proliferation or apoptosis between vehicle and ALW‐treated bone lesions (55.2% ± 2.7% Ki67+ nuclei vector vs 52.2% ± 3.1% Ki67+ nuclei ALW; *p* = 0.47 Mann–Whitney test; 0.68% ± 0.12% cleaved caspase 3+ nuclei vector vs 1.2% ± 0.24% cleaved caspase 3+ nuclei ALW; *p* = 0.20 Mann–Whitney test). We did observe a significant reduction in the number of TRAP+ bone proximal osteoclasts (Fig. 5*C*, arrows) in osteolytic lesions from ALW‐treated animals relative to vehicle‐treated controls (Fig. 5*C*, graph; *p* < 0.001). These data show that therapeutic targeting EphA2 impairs bone destruction in osteolytic breast cancer metastatic disease in vivo.

### EphA2 loss of function impairs tumor‐induced osteoclast differentiation in vitro

**Fig 6:**
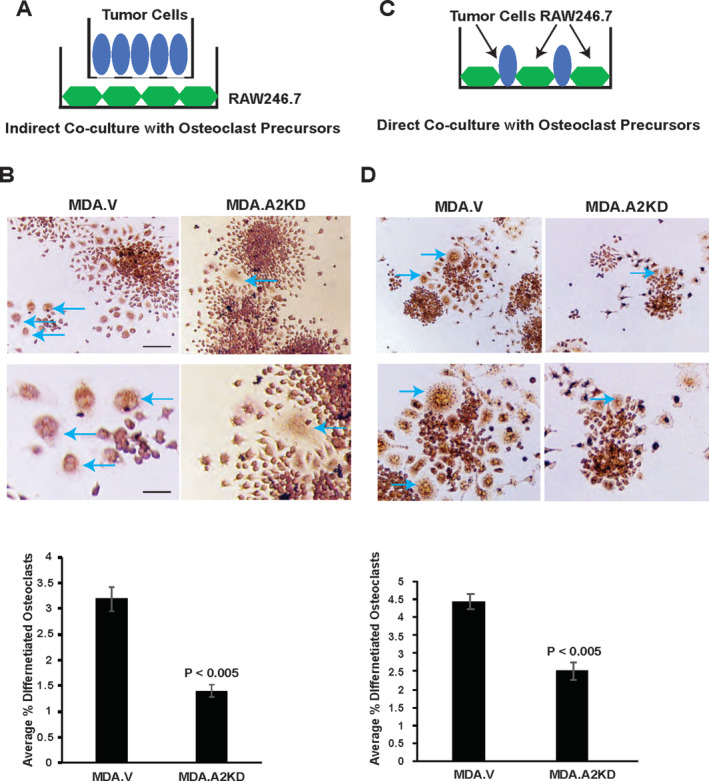
EphA2 loss of function in tumor cells impairs tumor‐induced osteoclast differentiation in coculture. (*A*) For indirect coculture assays, we seeded tumor cells in the upper chamber of transwells and seeded RAW264.7 osteoclast progenitor cells into the plate below, cultured for 6 days, and stained for tartrate‐resistant acid phosphatase (TRAP)+ osteoclasts (blue arrows). (*B*) Graph shows the % TRAP+ osteoclasts relative to the total number of cells for MDA.V and MDA.A2KD tumor cell cocultures (*p* < 0.005, Mann–Whitney test). Scale bar = 50 μm, upper panels; 25 μm, lower panels. (*C*) For direct coculture assays, we seeded tumor cells and progenitor cells into the same dish, cultured for 4 days, and stained for TRAP+ osteoclasts (blue arrows). (*D*) Graph shows % TRAP+ osteoclasts relative to the total number of cells for MDA.V and MDA.A2KD cocultures (*p* < 0.05, Mann–Whitney test). There were five to eight fields per condition from three independent experiments.

Although EphA2 loss of function impairs osteolysis, it is unclear if this effect is mediated by direct effects on osteoclast progenitors or indirectly through effects on other cell types, including tumor cells or other components of the bone microenvironment. Using a modified indirect coculture model (Fig. 6*A*), we investigated the ability of tumor cell EphA2 to induce osteoclast differentiation of progenitor cells. MDA.A2KD cells cocultured with RAW264.7 BALB/c‐derived macrophage‐like cells showed significantly reduced differentiation of TRAP+ osteoclasts (Fig. 6*B*, arrows) relative to MDA.V cells (Fig. 6*B*, graph; *p* < 0.005). We observed similar results for progenitor cells cocultured with  4T1.ΔC cells versus  4T1.V control cells (Supplementary Information Fig. *S3*A,B; *p* < 0.005). We also assessed progenitor cell differentiation to osteoclasts in direct coculture assays with MDA and  4T1 tumor models (Fig. 6C; Supplementary Information Fig. S3 *C*). The trend was similar to what we observed in indirect coculture assays, with significantly reduced TRAP+ osteoclast differentiation in MDA.A2KD and  4T1.ΔC cocultures relative to control MDA.V (Fig. 6*D*, arrows, graph; *p* < 0.005) and 4T1.V (Supplementary Information Fig. S3D, arrows, graph; *p* < 0.005), respectively. We did not observe any significant differences between the size of osteoclasts in vector control versus EphA2 loss of function tumor cells in coculture models (direct coculture average pixel area MDA.V 1758.5 ± 473 vs MDA.A2KD 1994 ± 826, arbitrary units, *p* = 0.583 Mann–Whitney test; indirect coculture average pixel area MDA.V 1721 ± 465 vs MDA.A2KD 1627 ± 270, arbitrary units, *p* = 0.702 Mann–Whitney test; direct coculture average pixel area  4T1.V 2551 ± 403 vs  4T1.ΔC 2321 ± 919, arbitrary units, *p* = 0.406 Mann–Whitney test; indirect coculture average pixel area 4T1.V 3346 ± 671 vs 4T1.ΔC 3978 ± 542, arbitrary units, *p* = 0.491 Mann–Whitney test). These data suggest EphA2 function in tumor cells is required for osteoclast differentiation in the context of breast cancer bone metastatic disease.

### EphA2 regulates osteoclast differentiation through an IL‐6–dependent mechanism

Data from coculture studies suggest that EphA2 induction of osteoclast differentiation by breast cancer cells involves a soluble factor(s) whose expression is regulated by EphA2. To identify differentially expressed soluble factors related to osteoclast differentiation and bone metastatic disease, we performed cytokine expression array analysis on conditioned media from 4T1.V versus  4T1.ΔC cells. We found that IL‐6 protein levels were significantly reduced in  4T1.ΔC versus  4T1.V controls (144 pg/ml 4T1.V vs 50 pg/ml 4T1.ΔC, 2.9‐fold decrease; *p* < 0.05 Mann–Whitney test).

**Fig 7:**
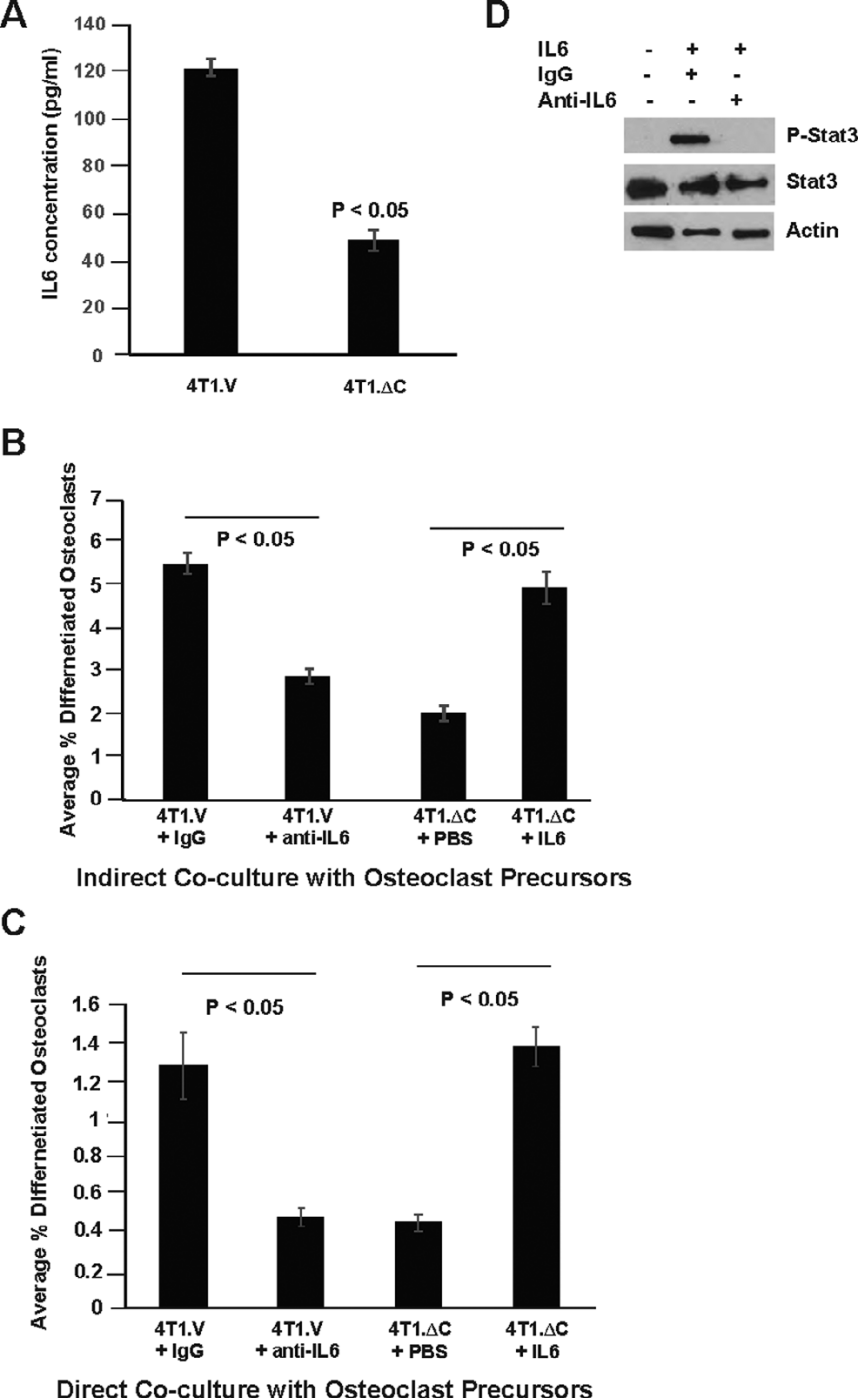
EphA2‐Dependent tumor induction of osteoclast differentiation requires IL‐6. (*A*) ELISA analysis for IL‐6 protein levels in conditioned media harvested from  4T1.ΔC cells relative to 4T1.V controls (*p* < 0.05, Mann–Whitney test). (*B*) Graph shows the % tartrate‐resistant acid phosphatase (TRAP)+ osteoclasts in indirect cocultures of osteoclast progenitors with  4T1.V + IgG, 4T1.V + anti‐mouse IL‐6 neutralizing antibody,  4T1.ΔC + PBS, and  4T1.ΔC + recombinant murine IL‐6. (*p* < 0.05, Mann–Whitney test). (*C*) Graph shows the % TRAP+ osteoclasts in direct cocultures of osteoclast progenitors with 4T1.V + IgG,  4T1.V + anti‐mouse IL‐6 neutralizing antibody,  4T1.ΔC + PBS, and  4T1.ΔC + recombinant murine IL‐6, (*p* < 0.05, Mann–Whitney test). There were five to eight fields per condition from three independent experiments. (*D*) Immunoblots show phosphorylated Stat3 levels in osteoclast progenitor cells treated with IL‐6 in the presence or absence of neutralizing anti‐mouse IL‐6 antibody. Uniform loading was confirmed by probing blots for total Stat3 and actin.

To validate data from the cytokine array, we analyzed 4T1.V and  4T1.ΔC conditioned media for IL‐6 levels by ELISA. We observed a 2.5‐fold decrease in IL‐6 protein levels in  4T1.ΔC‐conditioned medium versus control (Fig. 7*A*; *p* < 0.05), confirming reduced expression in EphA2 loss of function tumor cells. To determine the functional significance of IL‐6 reduction in tumor cell–induced osteoclast differentiation, we restored IL‐6 levels in  4T1.ΔC cells to levels comparable to those observed in control  4T1.V cells (e.g., added 75 pg to bring to 125 pg/ml) using recombinant murine IL‐6 and assessed osteoclast differentiation induced by tumor cells in indirect coculture assays. We also used a neutralizing anti‐mouse IL‐6 antibody to block IL‐6 function in cocultures with  4T1.V cells. Restoring IL‐6 levels rescued osteoclast differentiation induced by  4T1.ΔC cells to near control levels (Fig. 7*B*; *p* < 0.05), and neutralization of IL‐6 significantly inhibited osteoclast differentiation induced by 4T1.V cells (Fig. 7*B*; *p* < 0.05). We observed similar effects in direct coculture assays, where restoring IL‐6 levels in  4T1.ΔC cultures rescued osteoclast differentiation and blocking IL‐6 function in  4T1.V cultures inhibited osteoclast differentiation (Fig. 7*C*; *p* < 0.05). No significant differences in the size of osteoclasts were detected between groups (data not shown). We assessed phosphorylation of Stat3 as a surrogate marker for IL‐6 activity to confirm activity of recombinant IL‐6 and neutralizing activity of the anti‐mouse IL‐6 antibody (Fig. 7*D*). We also observed a significant reduction in IL‐6 expression for MDA.A2KD versus MDA.V control cells by ELISA (Supplementary Information Fig. S4A; *p* < 0.005). Restoring IL‐6 expression to control levels (e.g., added 3.3 ng to bring to 4 ng/ml) rescued osteoclast differentiation induced by MDA.A2KD tumor cells, and a neutralizing anti‐human IL‐6 antibody significantly inhibited osteoclast differentiation induced by MDA.V cells in indirect (Supplementary Information Fig. S4B; *p* < 0.05) and direct (Supplementary Information Fig. S4C; *p* < 0.05) coculture assays. No significant differences in the size of osteoclasts were detected between groups (data not shown). We confirmed recombinant human IL‐6 activity and neutralizing activity of anti‐human IL‐6 antibody using Stat3 phosphorylation as a surrogate marker (Supplementary Information Fig. S4D).

To validate data derived from RAW246.7 cells, we performed direct coculture assays with  4T1 cells and primary mouse bone marrow cells as the source for osteoclast progenitors (Supplementary Information Fig. S5A). Coculture with tumor cells resulted in osteoclast differentiation as determined by TRAP staining (Supplementary Information Fig. S5B, arrows). Coculture with  4T1.V cells resulted in a differentiation of a higher percentage of TRAP+ osteoclasts relative to coculture with  4T1.ΔC (Supplementary Information Fig. S5C, *p* < 0.05). Restoring IL‐6 expression to control levels rescued osteoclast differentiation induced by  4T1.ΔC tumor cells, and a neutralizing anti‐human IL‐6 antibody significantly inhibited osteoclast differentiation induced by  4T1.V cells (Supplementary Information Fig. S5C, *p* < 0.05). Together, these data suggest that elevated EphA2 expression in breast tumor cells promotes osteolytic disease in bone metastasis, at least in part, by upregulating IL‐6, which in turn enhances osteoclast differentiation to facilitate osteolysis.

## Discussion

The Eph receptor family and associated ephrin ligands play critical roles in many diverse cellular and disease processes.^11^ Breast cancer bone metastasis is predominantly osteolytic in nature, wherein osteoclasts are activated to cause bone degradation. Our study reveals that EphA2 functions in tumors to promote osteolysis of the bone via osteoclast differentiation, at least in part through regulation of IL‐6 production in tumor cells. Therefore, in the context of bone cell–tumor cell interactions, we hypothesized that blocking EphA2 will result in a decrease of osteoclast differentiation and activation, thus breaking the vicious cycle and offering an effective means to control bone metastasis. This hypothesis is supported by our in vivo studies showing the efficacy of molecular/genetic and pharmacologic inhibition of EphA2 in reducing osteolytic disease in breast cancer bone allograft and xenograft experimental metastasis models.

It will be of great interest to see if inhibition of EphA2 impairs endogenous metastasis to bone from an orthotopic primary tumor injection model, which will be particularly useful in determining which stages of the metastatic cascade are regulated by EphA2, including intravasation/extravasation, bone homing and colonization, and interaction with other components of the bone microenvironment such as inflammatory cells. These studies are ongoing. A relatively low number of metastatic samples are currently represented in the gene expression omnibus (GEO) data curated by the Human Cancer Metastasis database. We are in the process of identifying more cases for EphA2‐expression analyses to validate the clinical relevance of our findings. Although our studies with the pharmacologic inhibitor ALW‐II‐41‐27 provide proof‐of‐principle in terms of therapeutic targeting, this inhibitor is not suitable for clinical development. Adverse effects from ALW‐II‐41‐27 included lethargy, piloerection, and bruising at the injection site for mice treated with the inhibitor versus vehicle control. Future studies will test the efficacy of experimental therapeutics with the potential for clinical translation.

Recent studies identified roles for A‐class Eph receptors in bone homeostasis. EphA4 KO animals display craniosynostosis caused by dysregulation of proper osteogenic precursor‐cell migration and guidance,^44^ though we found no significant differences in *EPHA4* transcript levels in bone versus visceral metastases (data not shown). Ephrin‐A2 expressed in osteoclast precursors and EphA2 expressed in osteoblasts enhance osteoclastogenesis, while inhibiting osteoblast differentiation.^10^ Several Eph receptors, including EphA2, contribute to tumorigenesis and/or progression.^45^ Based on these data and our observed elevation of EphA2 in bone metastatic lesions relative to lesions from other metastatic sites, we hypothesized that EphA2 might play a key role in aberrant bone remodeling caused by increased expression in tumors where it mediates bone cell–tumor cell interactions. Breast cancer and multiple myeloma are associated with bone metastases that exhibit high levels of osteolysis,^46^, ^47^ whereas prostate cancers usually have higher levels of bone formation preceded by bone resorption,^48^ and all have alterations in Eph/ephrin signaling.^45^, ^49^ Indeed, suppression of EphA2 function in prostate cancer cells via overexpression of cytoplasmic deletion or kinase dead mutants impaired growth in bone.^20^ In our models of breast cancer, elevated EphA2 expression in tumor cells appears to be required for tumor‐induced osteolytic disease.

Our models support an indirect role for tumor‐expressed EphA2 in modulating osteoclast differentiation through regulation of IL‐6. IL‐6 plays an important role in osteoclast differentiation, bone homeostasis, and the vicious cycle of bone destruction in bone metastatic lesions.^50^ Moreover, evidence from studies of postinfectious irritable bowel syndrome and pulmonary fibrosis supports EphA2 regulation of IL‐6.^51^, ^52^ Although we cannot rule out a role for EphA2‐mediated reverse signaling through ephrin ligands expressed in osteoclast progenitors, comparable patterns of osteoclast differentiation in vitro for both direct and indirect tumor coculture, as well as comparable rescue of differentiation by exogenous IL‐6, support an indirect role. In addition, the fact that EphA2 KD in MDA‐MB‐231 tumor cells phenocopies expression of the dominant negative mutant capable of mediating reverse signaling in  4T1 tumor cells also provides support for this model. On the other hand, EphA2 or ephrin‐A2 KD in rat bone‐marrow–derived macrophages significantly impaired osteoclastogenesis in culture.^53^ Thus, it is possible that EphA2 and its ligands may interact and mediate osteoclastogenesis in breast cancer bone metastatic disease through functions in the host bone microenvironment. It will be interesting to determine if deletion of one or more ephrin‐A ligands in the host bone microenvironment affects osteolysis induced by EphA2‐overexpressing tumor cells, especially because ephrin‐A1 expression in normal bone has been reported, as well as elevated ephrin‐A1 expression in human osteosarcomas.^54^

Our studies revealed differences in cytokine production by cultured tumor cells (both human and murine) when EphA2 activity is inhibited. While we are in the process of validating differences in other candidate cytokines (e.g., GM‐CSF, M‐CSF, and/or SDF‐1α) between  4T1.V and  4T1.ΔC cells, we validated IL‐6 as a factor regulated by EphA2. IL‐6 is produced by several bone‐homing cancer cells, where it facilitates bone invasion and growth of tumor cells in the bone microenvironment.^55^ In addition, IL‐6 functions within bone to stimulate osteoclast activity and bone resorption,^56^, ^57^, ^58^ with IL‐6 KO mice showing resistance to experimental arthritis^59^ and treatment with an IL‐6 receptor antagonist reducing bone resorption in vivo.^60^ In the context of cancer, blockade of IL‐6 has been shown to reduce prostate cancer and breast cancer–induced bone destruction.^61^, ^62^, ^63^ It should be noted, however, that some studies have reported that adding IL‐6 directly to osteoclast progenitor cultures inhibits osteoclast differentiation by inhibition of RANK signaling pathways.^64^ Other studies have reported that the combination of TNFα and IL‐6 promoted osteoclast differentiation from bone marrow–derived macrophages.^65^ It is possible that the positive and negative effects of IL‐6 on osteoclastogenesis are influenced by other cytokines present in the microenvironment. It will be of great interest to determine the role other cytokines differentially regulated by EphA2 in tumor cells play in modulating osteoclast differentiation and function.

Though our models support a role for EphA2‐mediated IL‐6 osteoclast differentiation and osteolysis, we do not yet know if this regulatory pathway also mediates breast cancer cell homing to bone. It will be interesting to identify additional processes in the bone metastatic cascade regulated by EphA2/IL‐6, possibly in cooperation with other cytokines, as well as the molecular mechanism(s) that link EphA2 with IL‐6 expression or secretion. It will also be interesting to compare inflammatory cell populations, including macrophages, T cells, and other relevant populations and subpopulations, in EphA2‐defective versus control bone metastases in vivo.

Although the role of EphA2 in breast cancer and visceral metastasis has been well‐established, it is crucial to perform preclinical studies in clinically relevant models of metastasis at different anatomic sites. Indeed, the fact that EphA2 loss of function in the primary tumor within the mammary microenvironment impairs proliferation and/or survival^29^, ^40^ but has no effect on tumor cell proliferation in the bone microenvironment, highlights the importance of systematic testing in multiple models that reflect the spectrum of tumor progression in relevant microenvironments. Our data support the rationale for continued investigation into targeting cell–cell interactions via Eph receptors and ligands as a way to treat osteolytic bone disease that would offer the potential for increased therapeutic options available to patients suffering from this painful disease, particularly in light of a recent phase 1/2 clinical trial that reported that the combination of dasatinib and zoledronic acid was well‐tolerated and produced positive responses in bone for patients with hormone receptor–positive tumors.^27^ Bisphosphonates like zolendronic acid are the current standard of care for blocking tumor‐induced bone disease. Thus, the most likely clinical application of EphA2 inhibitors will involve a combination therapy with bisphosphonates. Because dasatinib targets many kinases, it will be of great interest to test more specific pharmacologic inhibitors of EphA2 for efficacy in treating bone metastatic disease, as well as potential synergy with bisphosphonates and other drugs that target osteoclast function.

In conclusion, this study reveals a novel function for EphA2 signaling in tumor cell–bone cell interactions involved in osteoclastogenesis and osteolysis associated with breast cancer metastasis, in part through regulation of cytokines such as IL‐6. Furthermore, our data provide preclinical support for targeting EphA2 in advanced‐stage breast cancer disease associated with bone metastasis to disrupt the vicious cycle.

## AUTHOR CONTRIBUTIONS

**David Vaught:** Conceptualization; data curation; formal analysis; investigation; methodology; validation; visualization; writing‐original draft. **Alyssa Merkel:** Formal analysis; investigation; methodology; writing‐review & editing. **Conor Lynch:** Resources. **James Edwards:** Data curation; formal analysis; methodology; resources; software; writing‐review & editing. **Mohammad Tantawy:** Data curation; formal analysis; methodology; software; writing‐review & editing. **Timothy Hilliard:** Data curation; formal analysis; investigation; methodology. **Shan Wang:** Conceptualization; data curation; methodology; visualization. **Todd Peterson:** Data curation; methodology; resources; software. **Rachelle Johnson:** Conceptualization; formal analysis; investigation; resources; writing‐review & editing. **Julie Sterling:** Formal analysis; investigation; methodology; resources; supervision; writing‐review & editing. **Dana Brantley‐Sieders:** Conceptualization; data curation; formal analysis; funding acquisition; investigation; methodology; project administration; supervision; validation; visualization; writing‐original draft; writing‐review & editing.

## Authors' roles

Study design: DBV and DBS. Study conduct: DBV, ARM, MNT, SW, TH, JAS, and DBS. Data collection: DBV, ARM, MNT, SW, TH, and DBS. Data analysis: DBV, ARM, CCL, JE, MNT, SW, TP, RWJ, JAS, and DBS. Data interpretation: DV, ARM, JE, RAW, JAS, and DBS. Drafting manuscript: DBV and DBS. Revising manuscript content: JE, MNT, RAW, JAS, and DBS. Approving final version of manuscript: DBV, ARM, CCL, JE, MNT, TH, SW, TP, RWJ, JAS, and DBS. DBS takes responsibility for the integrity of the data analysis.

## Conflicts of Interest

The authors declare no conflicts of interest.

### PEER REVIEW

The peer review history for this article is available at https://publons.com/publon/10.1002/jbm4.10465.

## Supporting information


**Figure S1** EphA2 expression and loss of function in MDA‐MB‐231 intratibial xenograft tumor model. (A) Immunohistochemistry staining for EphA2 was performed on MDA‐MB‐231 intratibial xenografts, showing expression in tumor cells embedded within the bone marrow space (arrows) as well as in associated blood vessel endothelium (*). Boxes in upper panels indicate areas magnified and presented in lower panels. Scale bar = 200 μm top left panel, 100 μm top right panel, 50 μm bottom panels. (B) Graph shows transcript levels of *EPHA2* in MDA‐MB‐231 cells stably expressing *ephA2* shRNA (MDA.A2KD) cells relative to vector controls (MDA.V, *p* < 0.05, Mann–Whitney test). Immunoblots show protein expression of EphA2 in MDA.A2KD cells relative to MDA.V controls. Uniform loading was confirmed by probing blots for actin. (D) Digital X‐rays of contralateral control limbs injected with PBS (mock) 3 weeks after injection relative to limbs injected with vector control tumor cells (MDA.V; arrows indicate osteolytic lesions. N = 9 to 11 per condition animals analyzed in three independent experiments.

**Figure S2** EphA2 expression and function in 4 T1 intratibial allograft tumor model. (A) Immunohistochemistry staining for EphA2 was performed on 4 T1 intratibial allografts, revealing expression in tumor cells embedded within the bone matrix (arrows) as well as in associated blood vessel endothelium (*). Boxes in upper panels indicate areas magnified and presented in lower panels. Scale bar = 200 μm top left panel, 100 μm top right panel, 50 μm bottom panels. (B) Immunoblot analysis for phosphorylated EphA2 activity in 4 T1 vector control (4 T1.V) versus 4 T1 cells expressing a truncated, dominant negative EphA2 receptor variant lacking the intracellular domain (4 T1.ΔC) in response to recombinant, soluble ephrin‐A1‐Fc (EfnA1‐Fc) ligand stimulation. Uniform loading was confirmed by probing blots for total EphA2 and actin. (C) Tissue sections from 4 T1.V and 4 T1.ΔC bone tumors were stained for Ki67 (arrows) to mark proliferating cells (not significant, Mann–Whitney test). Scale bar = 200 μm. (D) Sections from 4 T1.V and 4 T1.ΔC bone tumors were stained for cleaved caspase 3 (Cl. Caspase 3, arrows) to mark apoptotic cells (not significant, Mann–Whitney test). Scale bar = 200 μm. N = tissue sections from 5 independent animals/genotype, 4 independent 20X fields per tumor.

**Figure S3** EphA2 loss of function in tumor cells impairs tumor‐induced osteoclast differentiation in co‐culture. (A) For indirect co‐culture assays, we seeded tumor cells in the upper chamber of transwells and seeded osteoclast precursors into the plate below, cultured for six days, and stained for TRAP+ osteoclasts (blue arrows) (B) Graph shows the percent of TRAP+ osteoclasts relative to the total number of cells for 4 T1.V and 4 T1.ΔC tumor cell co‐cultures (*p* < 0.005, Mann–Whitney test). Scale bar = 50 μm upper panels and 25 μm lower panels. (C) For direct co‐culture assays, we seeded tumor cells and osteoclast precursors into the same dish, cultured for four days, and stained TRAP+ osteoclast (blue arrows). (D) Graph shows the percent of TRAP+ osteoclasts relative to the total number of cells for 4 T1.V and 4 T1.ΔC co‐cultures (p < 0.005, Mann–Whitney test). TC = tumor cells. OCP = osteoclast precursors cells. N = 5 to 8 fields/condition from 3 independent experiments.

**Figure S4** EphA2‐dependent tumor induction of osteoclast differentiation requires IL‐6. (A) ELISA analysis for IL‐6 protein in conditioned media harvested from MDA.A2KD cells relative to MDA.V controls of tumor cell conditioned medium (*p* < 0.005, Mann–Whitney test). (B) Indirect co‐cultures with osteoclast progentiors and MDA.A2KD + PBS, MDA.A2KD + recombinant human IL‐6, MDA.V + IgG, and MDA.V + anti‐human IL‐6 neutralizing antibody. Blue arrows show TRAP+ osteoclasts. Scale bar = 25 μm. (C) Graph shows the percent TRAP+ positive osteoclasts in indirect co‐cultures of osteoclast progenitors with MDA.V + IgG, MDA.V + anti‐human IL‐6 neutralizing antibody, MDA.A2KD + PBS, and MDA.A2KD + recombinant human IL‐6. (*p* < 0.05; Mann–Whitney test). (D) Graph shows the percent TRAP+ osteoclasts in direct co‐cultures of osteoclast progenitors with MDA.V + IgG, MDA.V + anti‐human IL‐6 neutralizing antibody, MDA.A2KD + PBS, and MDA.A2KD + recombinant human IL‐6, (p < 0.05; Mann–Whitney test). N = 5 to 8 fields/condition from 3 independent experiments. (E) Immunoblots show phosphorylated Stat3 levels in osteoclast progentior cells treated with human IL‐6 in the presence or absence of neutralizing anti‐human IL‐6 antibody. Uniform loading was confirmed by probing blots for total Stat3 and actin.

**Figure S5** EphA2‐dependent tumor induction of osteoclast differentiation from primary bone marrow progenitor cells requires IL‐6. (A) For direct co‐culture assays, we seeded tumor cells and primary mouse bone marrow (PMBM) cells into the same dish, cultured for eight days, and stained plates for differentiated, TRAP+ osteoclasts. (B) Graph shows the percent of TRAP+ osteoclasts (blue arrows) relative to the total number of cells in co‐cultures with 4 T1.V and 4 T1.ΔC tumor cells. Scale bar = 50 μm. (C) Graph shows the percent TRAP+ osteoclasts in direct co‐cultures of osteoclast progenitors with 4 T1.V + IgG, 4 T1.V + anti‐mouse IL‐6 neutralizing antibody, 4 T1.ΔC + PBS, and 4 T1.ΔC + recombinant murine IL‐6, (*p* < 0.05; Mann–Whitney test). N = 9.
