# Supplementary figures and images for: EphA2 Is a Clinically Relevant Target for Breast Cancer Bone Metastatic Disease
Source: JBMR Plus. 2021 Mar 9;5(4):e10465. doi: 10.1002/jbm4.10465 (PMC8046157; doi:10.1002/jbm4.10465)

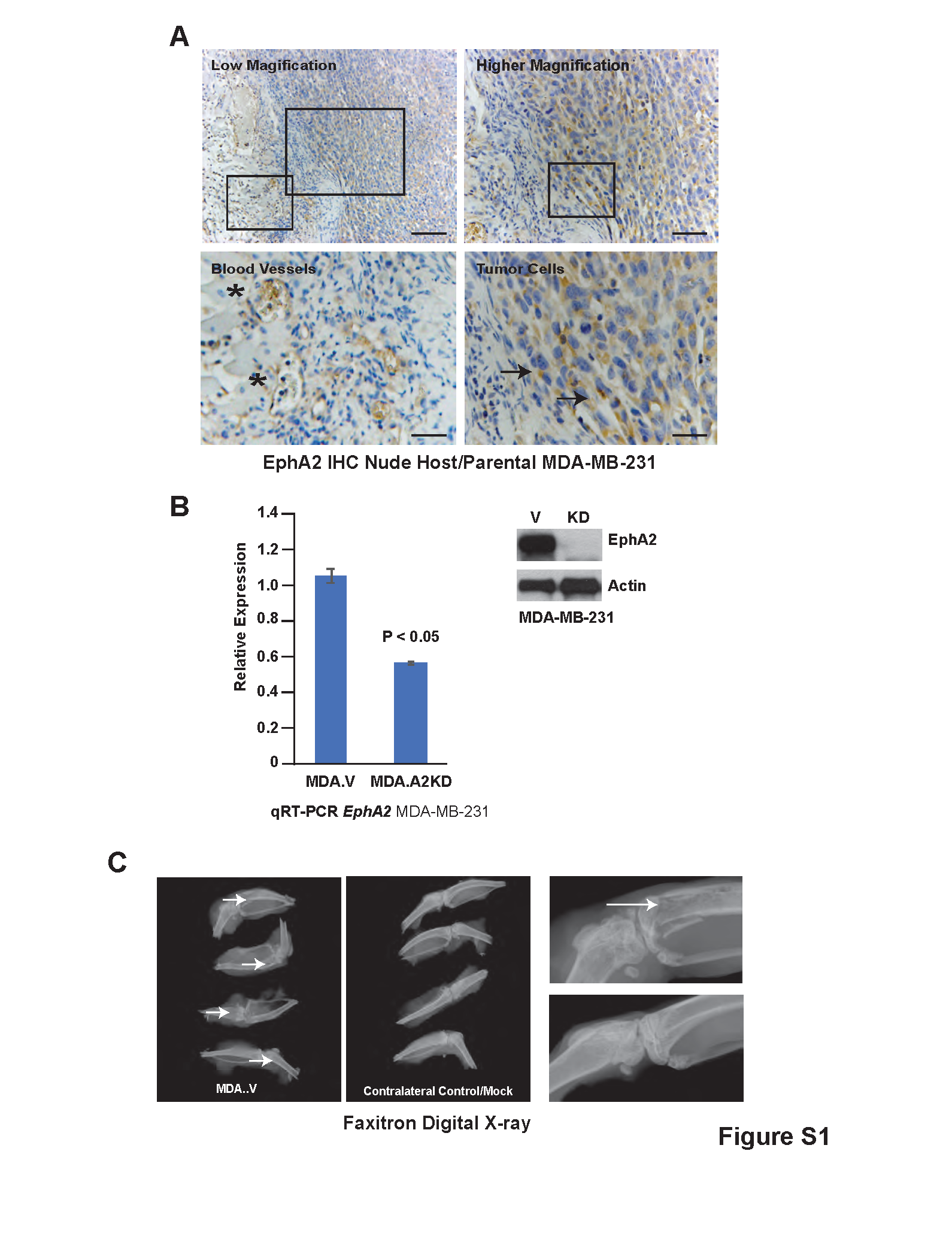

Supplement: Supplementary file 1 — Figure S1 EphA2 expression and loss of function in MDA‐MB‐231 intratibial xenograft tumor model. (A) Immunohistochemistry staining for EphA2 was performed on MDA‐MB‐231 intratibial xenografts, showing expression in tumor cells embedded within the bone marrow space (arrows) as well as in associated blood vessel endothelium (*). Boxes in upper panels indicate areas magnified and presented in lower panels. Scale bar = 200 μm top left panel, 100 μm top right panel, 50 μm bottom panels. (B) Graph shows transcript levels of EPHA2 in MDA‐MB‐231 cells stably expressing ephA2 shRNA (MDA.A2KD) cells relative to vector controls (MDA.V, p < 0.05, Mann–Whitney test). Immunoblots show protein expression of EphA2 in MDA.A2KD cells relative to MDA.V controls. Uniform loading was confirmed by probing blots for actin. (D) Digital X‐rays of contralateral control limbs injected with PBS (mock) 3 weeks after injection relative to limbs injected with vector control tumor cells (MDA.V; arrows indicate osteolytic lesions. N = 9 to 11 per condition animals analyzed in three independent experiments. [file JBM4-5-e10465-s003.tiff]

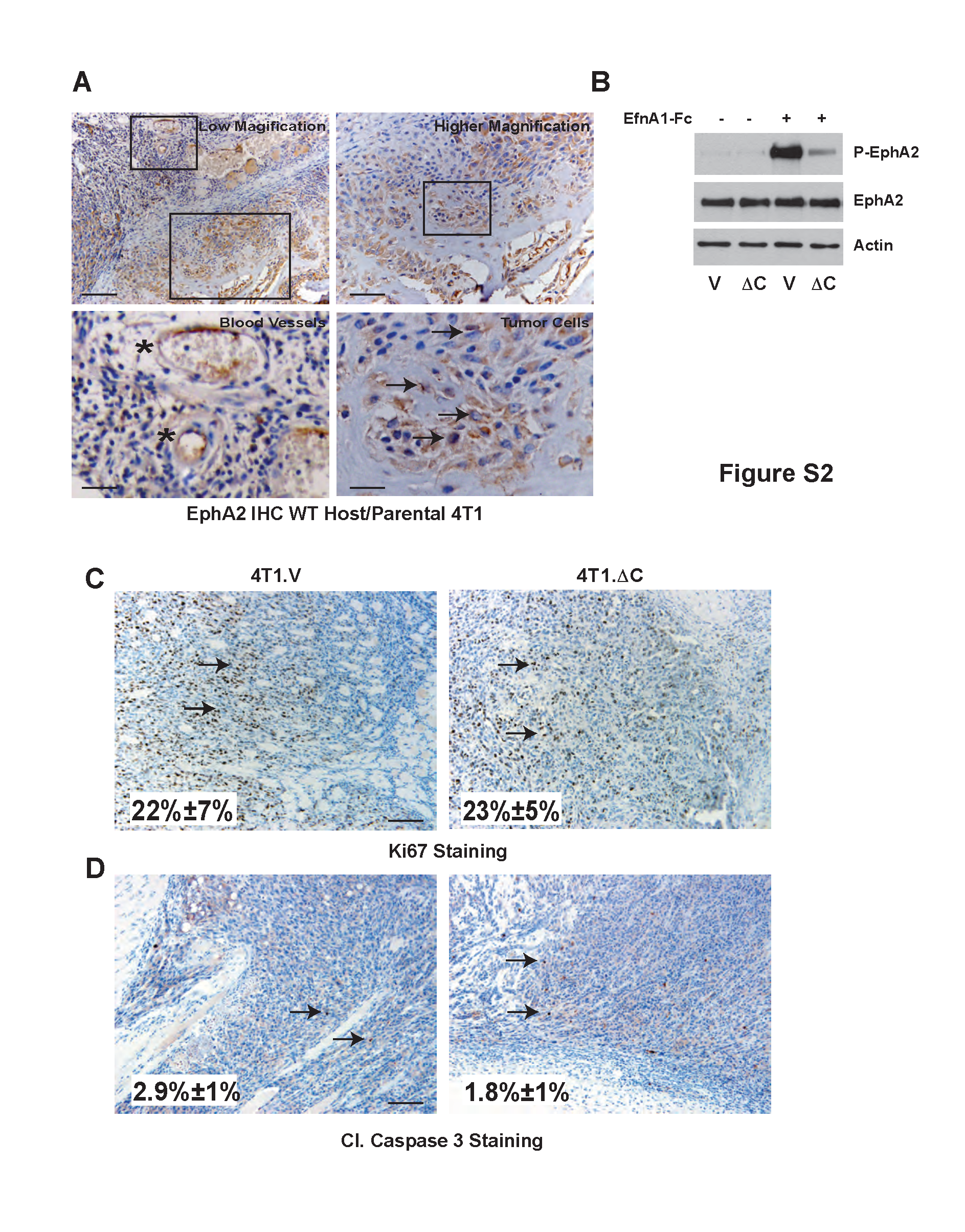

Supplement: Supplementary file 2 — Figure S2 EphA2 expression and function in 4 T1 intratibial allograft tumor model. (A) Immunohistochemistry staining for EphA2 was performed on 4 T1 intratibial allografts, revealing expression in tumor cells embedded within the bone matrix (arrows) as well as in associated blood vessel endothelium (*). Boxes in upper panels indicate areas magnified and presented in lower panels. Scale bar = 200 μm top left panel, 100 μm top right panel, 50 μm bottom panels. (B) Immunoblot analysis for phosphorylated EphA2 activity in 4 T1 vector control (4 T1.V) versus 4 T1 cells expressing a truncated, dominant negative EphA2 receptor variant lacking the intracellular domain (4 T1.ΔC) in response to recombinant, soluble ephrin‐A1‐Fc (EfnA1‐Fc) ligand stimulation. Uniform loading was confirmed by probing blots for total EphA2 and actin. (C) Tissue sections from 4 T1.V and 4 T1.ΔC bone tumors were stained for Ki67 (arrows) to mark proliferating cells (not significant, Mann–Whitney test). Scale bar = 200 μm. (D) Sections from 4 T1.V and 4 T1.ΔC bone tumors were stained for cleaved caspase 3 (Cl. Caspase 3, arrows) to mark apoptotic cells (not significant, Mann–Whitney test). Scale bar = 200 μm. N = tissue sections from 5 independent animals/genotype, 4 independent 20X fields per tumor. [file JBM4-5-e10465-s001.tiff]

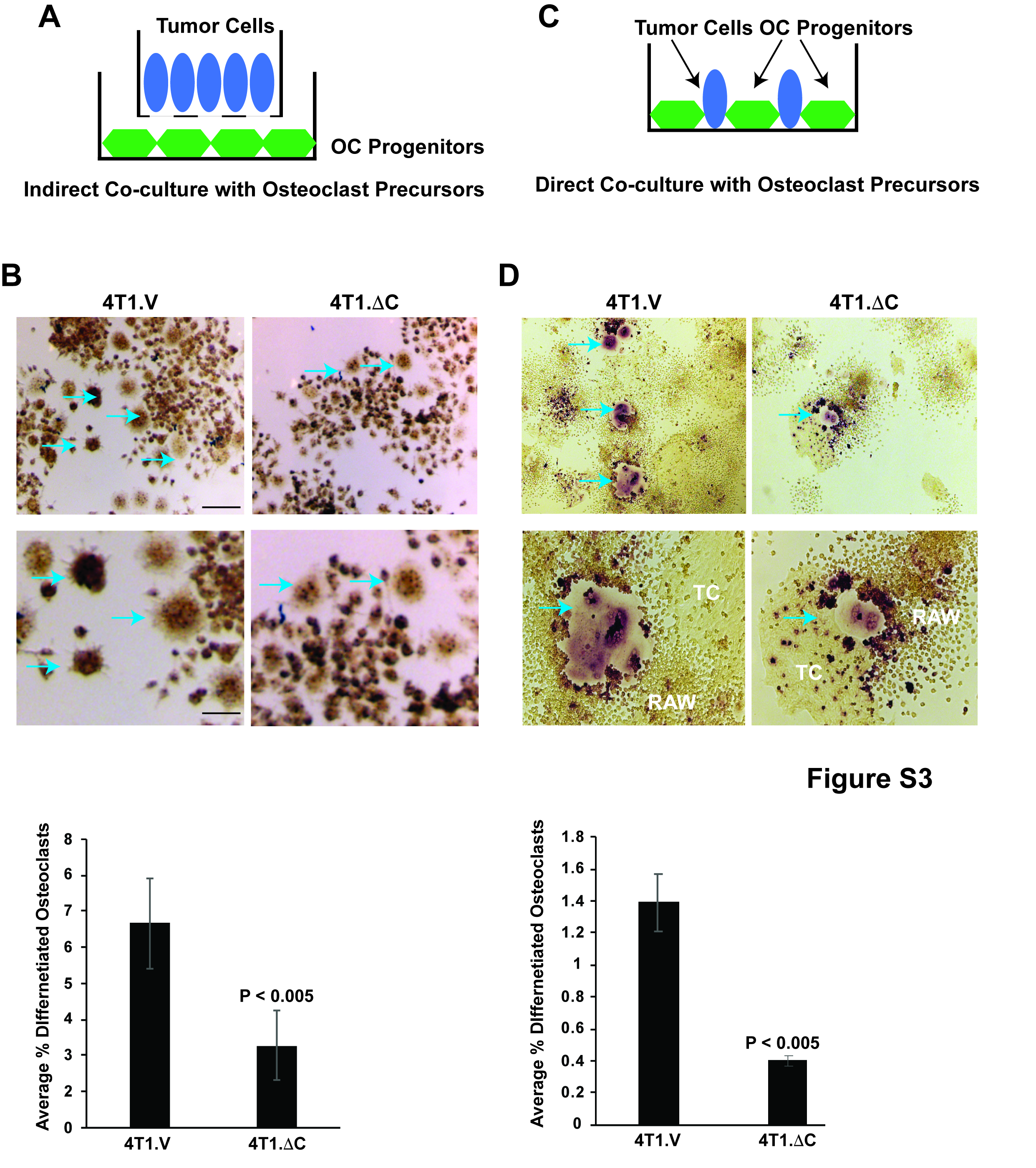

Supplement: Supplementary file 3 — Figure S3 EphA2 loss of function in tumor cells impairs tumor‐induced osteoclast differentiation in co‐culture. (A) For indirect co‐culture assays, we seeded tumor cells in the upper chamber of transwells and seeded osteoclast precursors into the plate below, cultured for six days, and stained for TRAP+ osteoclasts (blue arrows) (B) Graph shows the percent of TRAP+ osteoclasts relative to the total number of cells for 4 T1.V and 4 T1.ΔC tumor cell co‐cultures (p < 0.005, Mann–Whitney test). Scale bar = 50 μm upper panels and 25 μm lower panels. (C) For direct co‐culture assays, we seeded tumor cells and osteoclast precursors into the same dish, cultured for four days, and stained TRAP+ osteoclast (blue arrows). (D) Graph shows the percent of TRAP+ osteoclasts relative to the total number of cells for 4 T1.V and 4 T1.ΔC co‐cultures (p < 0.005, Mann–Whitney test). TC = tumor cells. OCP = osteoclast precursors cells. N = 5 to 8 fields/condition from 3 independent experiments. [file JBM4-5-e10465-s005.tif]

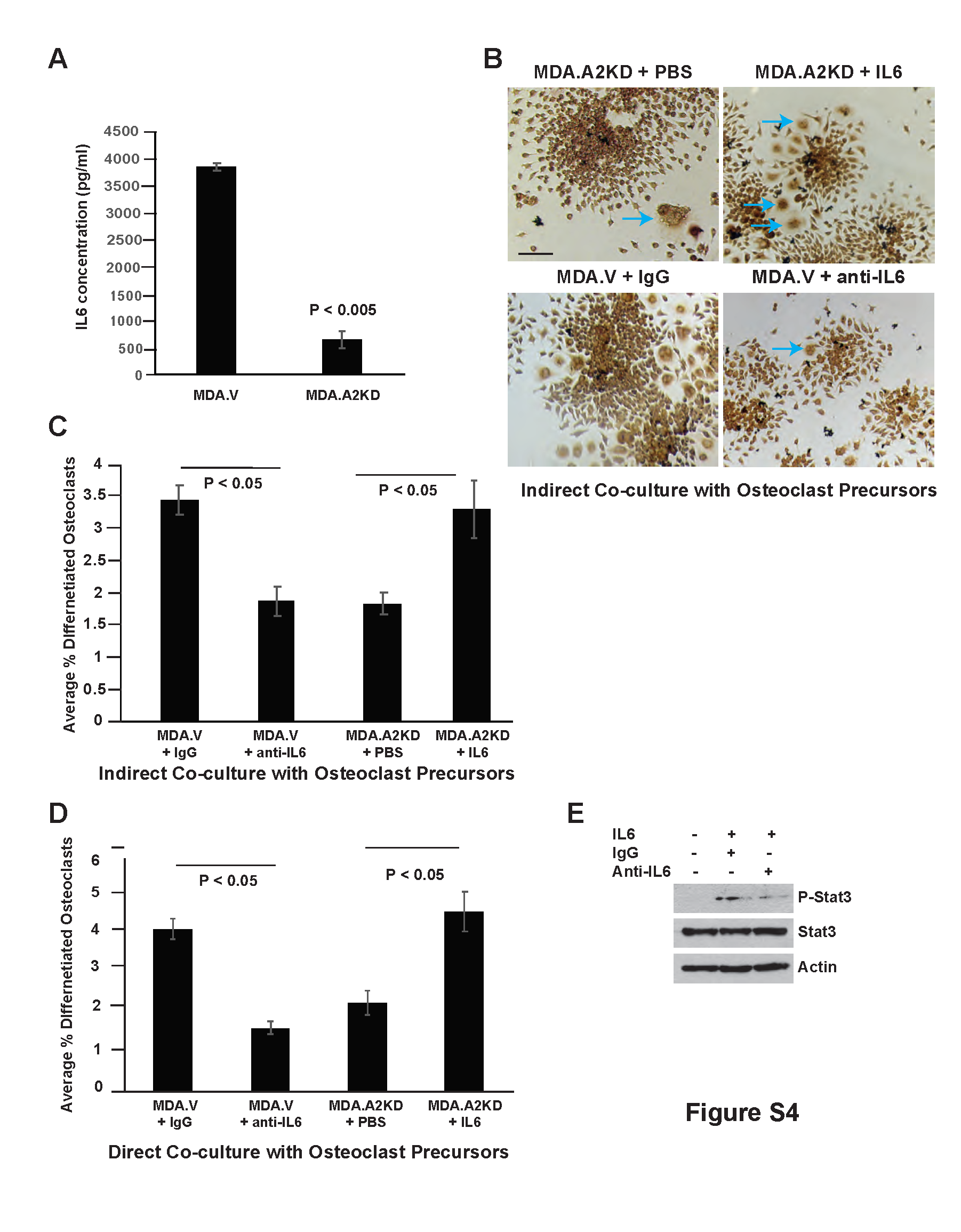

Supplement: Supplementary file 4 — Figure S4 EphA2‐dependent tumor induction of osteoclast differentiation requires IL‐6. (A) ELISA analysis for IL‐6 protein in conditioned media harvested from MDA.A2KD cells relative to MDA.V controls of tumor cell conditioned medium (p < 0.005, Mann–Whitney test). (B) Indirect co‐cultures with osteoclast progentiors and MDA.A2KD + PBS, MDA.A2KD + recombinant human IL‐6, MDA.V + IgG, and MDA.V + anti‐human IL‐6 neutralizing antibody. Blue arrows show TRAP+ osteoclasts. Scale bar = 25 μm. (C) Graph shows the percent TRAP+ positive osteoclasts in indirect co‐cultures of osteoclast progenitors with MDA.V + IgG, MDA.V + anti‐human IL‐6 neutralizing antibody, MDA.A2KD + PBS, and MDA.A2KD + recombinant human IL‐6. (p < 0.05; Mann–Whitney test). (D) Graph shows the percent TRAP+ osteoclasts in direct co‐cultures of osteoclast progenitors with MDA.V + IgG, MDA.V + anti‐human IL‐6 neutralizing antibody, MDA.A2KD + PBS, and MDA.A2KD + recombinant human IL‐6, (p < 0.05; Mann–Whitney test). N = 5 to 8 fields/condition from 3 independent experiments. (E) Immunoblots show phosphorylated Stat3 levels in osteoclast progentior cells treated with human IL‐6 in the presence or absence of neutralizing anti‐human IL‐6 antibody. Uniform loading was confirmed by probing blots for total Stat3 and actin. [file JBM4-5-e10465-s004.tiff]

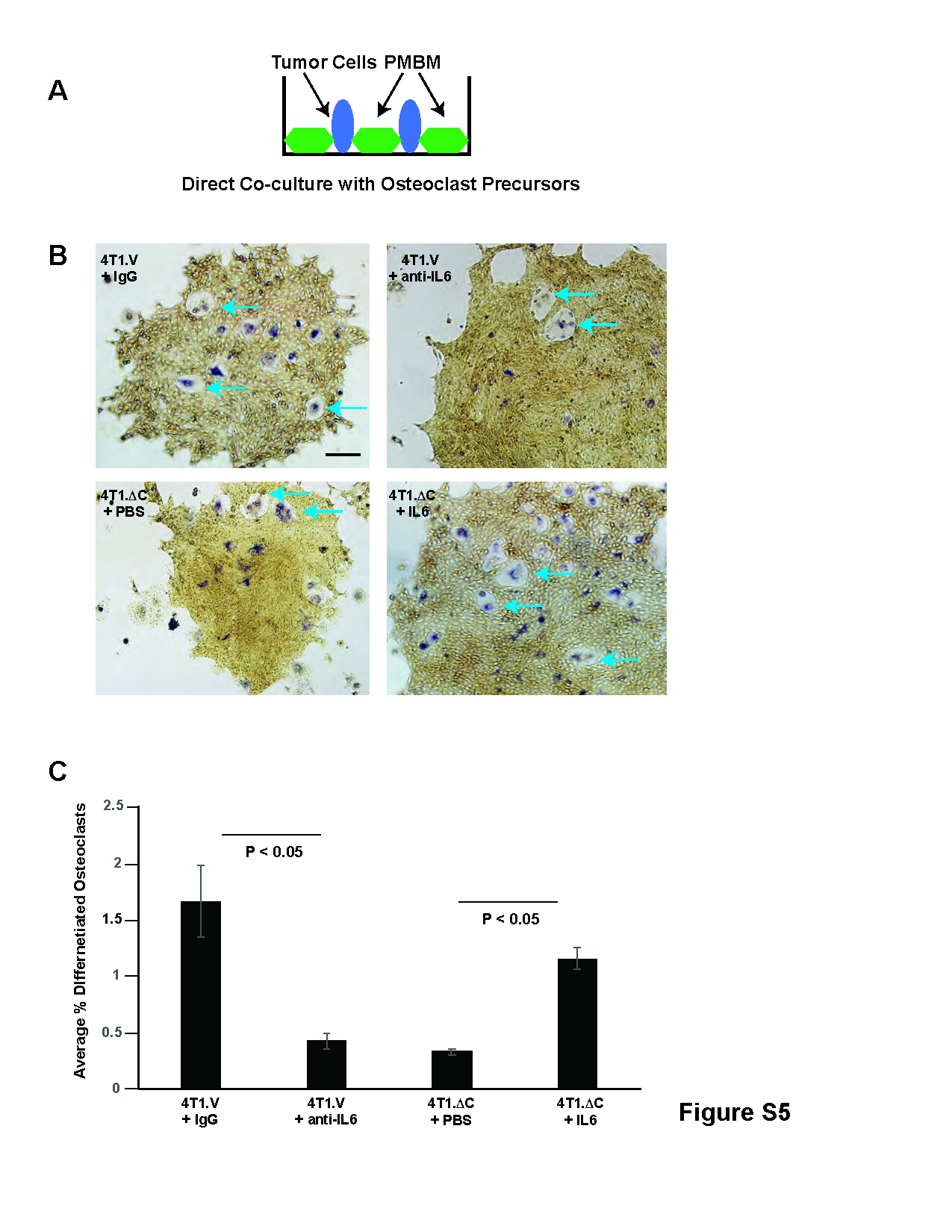

Supplement: Supplementary file 5 — Figure S5 EphA2‐dependent tumor induction of osteoclast differentiation from primary bone marrow progenitor cells requires IL‐6. (A) For direct co‐culture assays, we seeded tumor cells and primary mouse bone marrow (PMBM) cells into the same dish, cultured for eight days, and stained plates for differentiated, TRAP+ osteoclasts. (B) Graph shows the percent of TRAP+ osteoclasts (blue arrows) relative to the total number of cells in co‐cultures with 4 T1.V and 4 T1.ΔC tumor cells. Scale bar = 50 μm. (C) Graph shows the percent TRAP+ osteoclasts in direct co‐cultures of osteoclast progenitors with 4 T1.V + IgG, 4 T1.V + anti‐mouse IL‐6 neutralizing antibody, 4 T1.ΔC + PBS, and 4 T1.ΔC + recombinant murine IL‐6, (p < 0.05; Mann–Whitney test). N = 9. [file JBM4-5-e10465-s002.tiff]
